# Outcomes of Left Atrial Appendage Occlusion in Hispanic/Latino Patients: Insights From the National Inpatient Sample

**DOI:** 10.1002/clc.70152

**Published:** 2025-05-14

**Authors:** Mashli Fleurestil, Ayush Mohan, Ian Ergui, Jacklyn Samaha, Rosario Colombo, Raul Mitrani, Eduardo de Marchena, Pedro Villablanca, Jose Wiley, Yiannis S. Chatzizisis, Pedro Cox

**Affiliations:** ^1^ Department of Medicine, Division of Internal Medicine University of Miami Miller School of Medicine Miami Florida USA; ^2^ Department of Cardiology Jackson Memorial Hospital Miami Florida USA; ^3^ Department of Medicine, Division of Cardiology University of Miami Miller School of Medicine Miami Florida USA; ^4^ Department of Medicine, Division of Cardiology Henry Ford Health Detroit Michigan USA; ^5^ Section of Cardiology, Department of Medicine Tulane University New Orleans Los Angeles USA

**Keywords:** health disparities, Hispanic/Latino, LAAO, major bleeding, vascular complications, venous thromboembolism

## Abstract

**Background:**

Left atrial appendage occlusion (LAAO) is an established therapy for stroke prevention in non‐valvular atrial fibrillation (NVAF), but outcomes in Hispanic populations remain underexplored.

**Objective:**

The objective of our study was to evaluate the inpatient outcomes of Hispanic patients undergoing LAAO as compared to non‐Hispanic white patients.

**Methods:**

We conducted a retrospective cohort study using the National Inpatient Sample (NIS). From 157 434 LAAO hospitalizations identified, 133 517 were non‐Hispanic white and 6814 were Hispanic/Latino. The primary outcome was in‐hospital mortality.

**Results:**

Unadjusted odds in the Hispanic/Latino group were higher for mortality (OR 1.78, 95% CI 1.18−2.68, *p* 0.006), stroke (OR 1.64, 95% CI 1.26−2.14, *p* < 0.001), infectious complications (OR 3.89, 95% CI 3.03−4.99, *p* < 0.001), major bleeding (OR 1.22, 95% CI 1.11−1.33, *p* < 0.001), DVT/PE (OR 2.15, 95% CI 1.58−2.93, *p* < 0.001), and vascular complications (OR 1.81, 95% CI 0.53−0.93, *p* < 0.001). After adjusting for covariates and comorbidities, Hispanic/Latino patients had still greater odds of mortality (aOR 1.20, 95% CI 0.75−1.92, *p* 0.445), infectious complications (aOR 3.54, 95% CI 2.62−4.55, *p* < 0.001), and vascular complications (aOR 1.57, 95% CI 1.22−2.03, *p* < 0.001). Non‐Hispanic white patients had higher adjusted odds of pericardial effusion/tamponade (aOR 0.64, 95% CI 0.52−0.95, *p* 0.03), while Hispanic/Latino patients also had higher adjusted odds of cardiac arrest (aOR 1.99, 95% CI 1.15−3.42, *p* 0.46).

**Conclusion:**

Hispanic/Latino patients undergoing LAAO experience higher odds of infectious and vascular complications compared to non‐Hispanic white patients. These findings highlight the need to further investigate disparities in procedural outcomes.

## Graphical abstract

In Hispanic/Latino patients undergoing left atrial appendage occlusion (LAAO), both unadjusted and adjusted analyses reveal significantly higher odds of infectious and vascular complications compared to non‐Hispanic white patients.

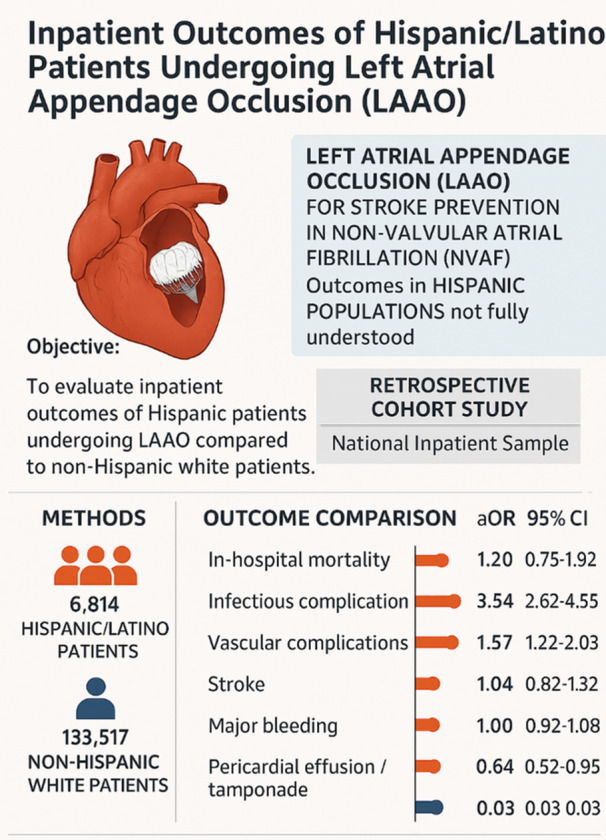

## 1 Introduction

Left atrial appendage occlusion (LAAO) is a therapeutic strategy for patients with non‐valvular atrial fibrillation (NVAF) that have increased risk of thromboembolic strokes and history of major bleeding [1, 2]. This treatment approach decreases the risk of stroke by eliminating a well‐known nidus of thrombi with an implantable device [3]. Randomized control trials have clearly demonstrated that LAAO with Watchman devices provide comparable stroke prevention to Warfarin while reducing bleeding risks [4]. While differences in utilization across ethnic groups have been well documented, often citing social determinants of health such as insurance coverage, associated costs, language barriers, and provider specific differences in patient selections as potential causes [5, 6, 7, 8, 9], there is a lack of sufficient data regarding the outcomes of the LAAO procedure in racial minority groups.

The National Cardiovascular Disease Registry (NCDR) LAAO Registry is the largest database in the United States, providing detailed insights into the use, outcomes, and safety of this intervention. However, an analysis of patient characteristics in the NCDR LAAO Registry reveals that Hispanic patients accounted for just 0.5% of the population during the first 3 years of the registry's existence [10]. Hispanic/Latino patients have also been shown to experience a higher frequency of embolic strokes even after controlling for social determinants of health [11]. Evidence also suggests a higher frequency of stroke recurrence in Hispanic patients, despite a lower incidence of disability compared to Black and White patients [12].

Despite this well‐documented trend of worse cardiovascular outcomes in Hispanic patients with atrial fibrillation, there is a lack of data on outcomes for Hispanic patients who undergo LAAO. Our objective was to evaluate in‐hospital outcomes for Hispanic patients following LAAO, and we hypothesized that these patients would experience higher rates of intraoperative bleeding, longer hospitalizations, and increased mortality, due to a combination of social determinants of health and provider specific differences.

## 2 Methods

### 2.1 Study Design

We performed a retrospective analysis using the National Inpatient Sample (NIS) database. The NIS, an extension of the Health care Cost and Utilization Project (HCUP), is funded by the Agency for Health care Research and Quality and is the largest inpatient database in the United States.

**Figure 1:**
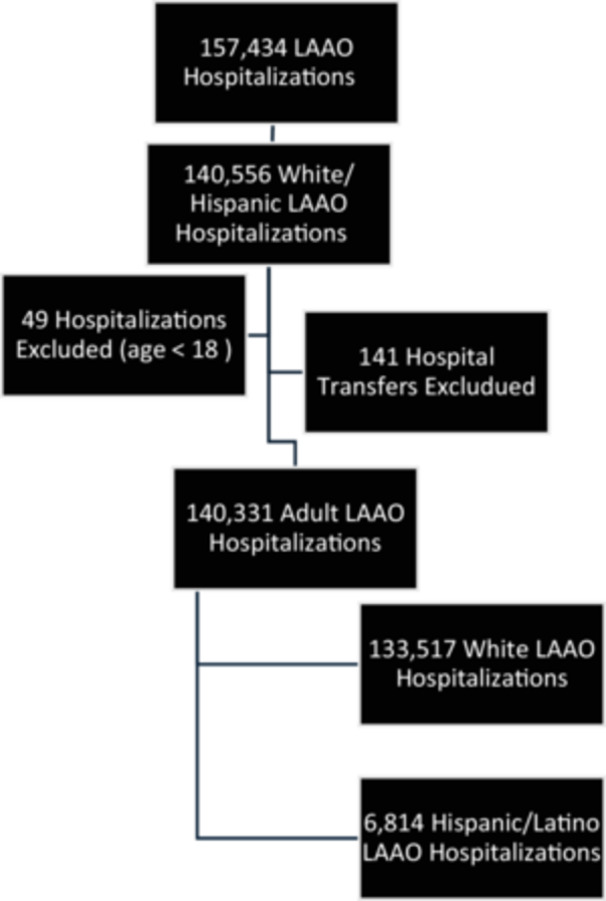
Flow chart detailing the selection of LAAO hospitalizations between 2011 and 2021.

The NIS data was queried from 2011 to 2021, which yielded 389 912 056 weighted cases. Relevant ICD‐9 and ICD‐10 codes, as listed in the supplemental materials (Supporting Information S1: Tables S1–S3), were used to identify patients with that were admitted for LAAO (*n* = 157 434). After isolating white and Hispanic/Latino patients (*n* = 140 556), identified by the uniform coding system used within the NIS, and excluding patients younger than 18 years of age and hospital transfers, a total of 140 331 LAAO hospitalizations were identified with 4.86% of them having Hispanic/Latino background (Figure 1).

The primary outcome measure was in hospital mortality. Secondary outcomes included pericardial effusion/tamponade, cardiac arrest, stroke complications, infectious complications, all major bleeding, DVT/PE, vascular complications, intubation, length of stay and cost of stay. For continuous and categorical variables, descriptive statistics were used. Mean and standard deviation are provided for categorical variables. For nonparametric continuous variables, the median and interquartile range (IQR) were reported, and categorical variables were given percentages. Pearson's chi‐square testing was performed for categorical variables, and parametric continuous variables were analyzed using independent samples *t*‐tests. For all non‐parametric continuous variables, the Mann−Whitney *U* test was employed. Statistical significance was confirmed by a *p* value of < 0.05 for contiguous and categorical variables. Adjusted odds ratios (aOR) and 95% confidence intervals with significant *p* values of < 0.001 were developed by using a binary logistic regression to adjust for comorbidities. Statistical analysis was performed using version 26.0 of SPSS statistical software (IBM corporation).

## 3 Results

**Figure 2:**
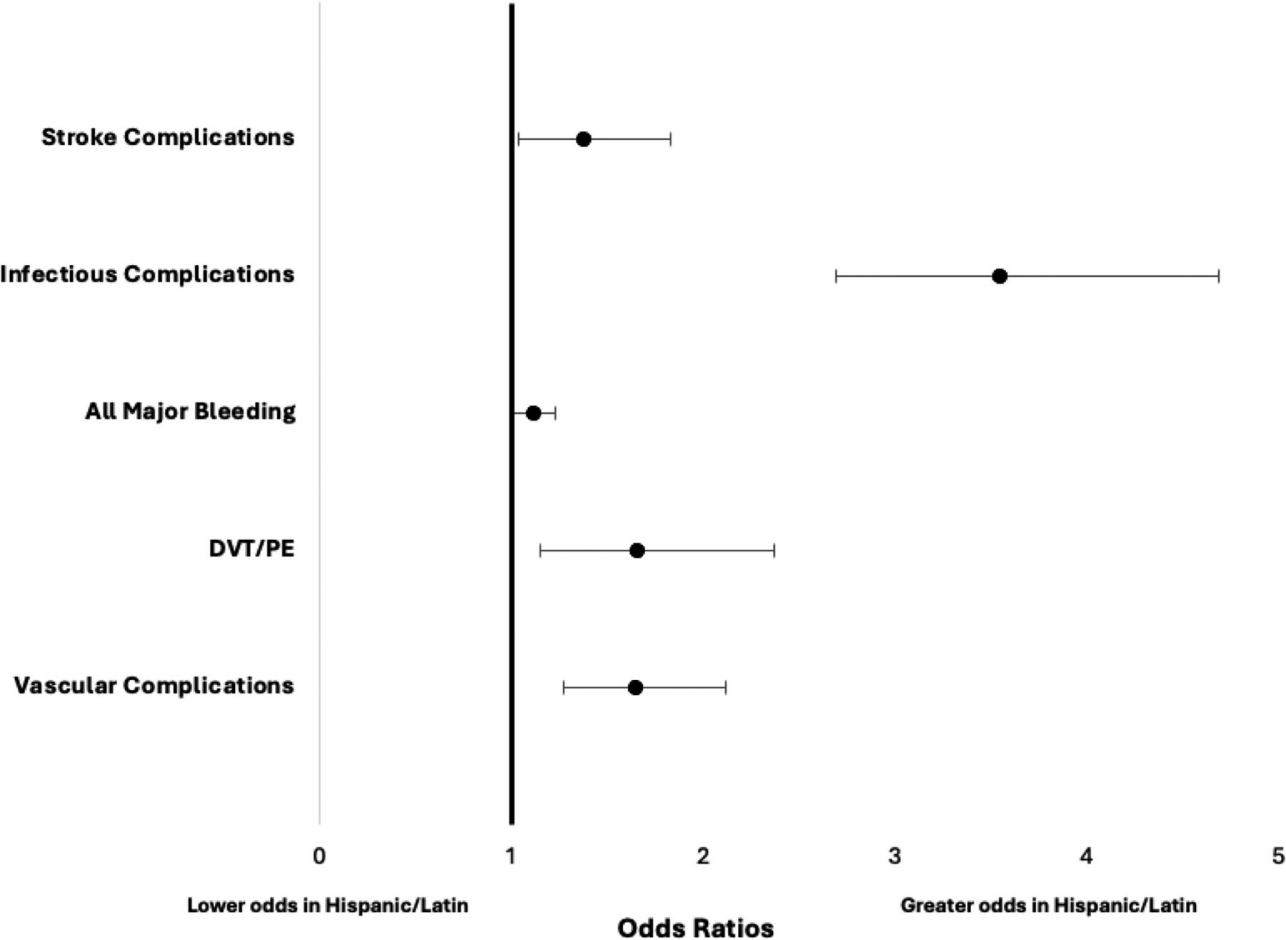
Adjusted in‐hospital outcomes and events in Hispanic/Latin versus White patients hospitalized for LAAO. Values > 1 indicate higher odds in patients Hispanic/Latin patients. LAAO, left atrial appendage occlusion.

Baseline characteristics of the patient sample are listed in Table 1. Of the patients represented in the 140 331 LAAO hospitalizations, 133 517 were white and 6814 were Hispanic/Latino. The mean age for the sample population was 75.3 in the white group compared to 73.4 in the Hispanic/Latino group. The gender distribution between the groups was 52 831 females (39.6%) 80 686 males (60.4%) in the white group, and 2949 (43.2%) female and 3865 (56.8%) male in the Hispanic/Latino group. Figure 2

**Table 1:** Baseline characteristics of patients admitted for LAAO.

Variable	White (*n* = 133 517)	Hispanic/Latino (*n* = 6814)	*p* value
Age	75.53 ± 8.27	73.48 ± 9.79	< 0.05*
Female	39.6% (52 831)	43.2% (2949)	< 0.05*
Elective admission	91.9% (122 479)	84.3% (5729)	< 0.05*
Hypertension	56.9% (75 007)	58.1% (3915)	0.082
Diabetes mellitus	32.5% (43 238)	46.1% (3140)	< 0.05*
Coronary artery disease	46.8% (62 342)	47.0% (3185)	0.729
Prior myocardial infarction	12.1% (16 195)	11.5% (785)	0.133
Chronic kidney disease	22.1% (29 413)	25.1% (1700)	< 0.05*
On hemodialysis	1.8% (2379)	6.2% (420)	< 0.05*
History of heart failure	36.8% (49 157)	39.8% (2715)	< 0.05*
Prior stroke	23.3% (31 171)	24.8% (1690)	< 0.05*
Tobacco use	39.1% (52 270)	32.1% (2190)	< 0.05*
Alcohol use	1.3% (1668)	1.0% (70)	0.111
Substance abuse	0.4% (495)	0.7% (45)	< 0.05*
Anemia	5.4% (7130)	5.0% (335)	0.140
Asthma	4.9% (6495)	6.3% (420)	< 0.05*
Chronic obstructive pulmonary disease	17.1% (22 651)	12.5% (850)	< 0.05*
Obese	18.0% (24,099)	18.6% (1270)	0.218
Peripheral artery disease	7.6% (9895)	5.2% (350)	< 0.05*
Obstructive sleep apnea	23.3% (31 164)	17.5% (1190)	< 0.05*
Hyperlipidemia	64.0% (83 625)	66.4% (4465)	< 0.05*
Malignancy history	16.8% (22 368)	10.0% (680)	< 0.05*
Chronic liver disease and cirrhosis	2.5% (3260)	4.7% (320)	< 0.05*

*Note:* Values are reported as mean ± standard deviation for continuous variables and percentage (number) for categorical variables. *p* ≤ 0.05 is considered significant (asterisk).

The Hispanic/Latino patients were overall younger and more of them identified as women. Non‐Hispanic white patients were more likely to have an elective admission (91.9% vs 43.2% in the Hispanic/Latino group). Regarding chronic conditions, Hispanic/Latino patients had a statistically significant higher incidence of diabetes mellitus (46.1%), chronic kidney disease (25.1%), more often underwent hemodialysis (6.2%), and had a history of heart failure (39.8%), stroke (24.8%), and asthma (6.3%). Non‐Hispanic white patients were found to have more chronic obstructive pulmonary disease (17.1%), peripheral arterial disease (7.6%), obstructive sleep apnea (23.3%), history of malignancy (16.8%), and chronic liver disease and cirrhosis (2.5%). Hispanic/Latino patients were found to have a higher incidence of substance use (0.7%). White patients, however, had an increased level of tobacco use (39.1%). There were no statistically significant differences between the groups for alcohol use and rate of obesity.

**Figure 3:**
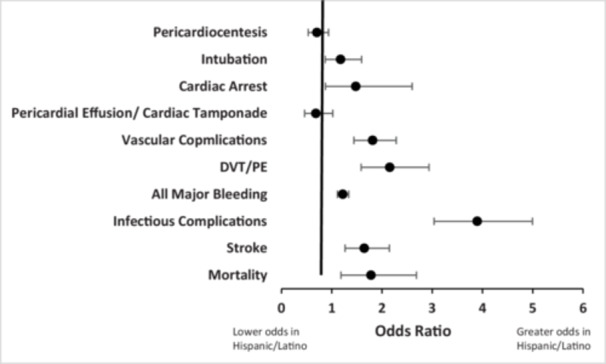
Unadjusted odds ratios for outcomes for LAAO hospitalizations. Outcomes reported as 95% confidence intervals. Values > 1 indicate higher odds in patients Hispanic/Latin patients. *p* value <0.001 was statistically significant.

The unadjusted in‐hospital outcomes for both groups are listed in Table 2. The incidence of mortality (0.3%), stroke complications (0.9%), infectious complications (1.1%), all major bleeding (7.6%), and post procedure DVT/PE (0.7%) was higher in the Hispanic/Latino group. Conversely, white patients had both decreased length (median 1.00 days, IQR 0 vs. median 1.00 days, IQR 1.00) and costs of stay (white median $109 486 IQR 84 767 vs. Hispanic median $145 276 IQR 104 065). Figure 3 presents the unadjusted outcomes as odds ratios. The unadjusted odds ratios for in hospital outcomes in the Hispanic/Latino group showed higher odds of mortality (OR 1.20, 95% CI 0.75−1.92, *p* 0.445), stroke (OR 1.64, 95% CI 1.26−2.14, *p* < 0.001), infectious complications (OR 3.89, 95% CI 3.03−4.99, *p* < 0.001), all major bleeding (OR 1.22, 95% CI 1.11−1.33, *p* < 0.001), DVT/PE (OR 2.15, 95% CI 1.58−2.93, *p* < 0.001), and vascular complications (OR 1.81, 95% CI 0.53−0.93, *p* < 0.001).

**Table 2:** Events and outcomes for LAAO hospitalizations.

Variable	White (*n* = 133 517)	Hispanic/Latino (*n* = 6814)	*p* value
Mortality	0.2% (265)	0.3% (20)	0.089
Pericardial effusion/tamponade	0.5% (713)	0.4% (25)	0.063
Cardiac arrest	0.1% (188)	0.2% (15)	0.093
Stroke complications	0.5% (674)	0.9% (60)	< 0.05*
Infectious complications^a^	0.3% (365)	1.1% (75)	< 0.05*
All major bleeding	6.3% (8415)	7.6% (515)	< 0.05*
DVT/PE	0.3% (400)	0.7% (45)	< 0.05*
Vascular complication	0.6% (843)	1.2% (80)	< 0.05*
Intubation	0.6% (739)	0.7% (45)	0.248
Length of stay^b^ (days)	1.00 (IQR 0)	1.00 (IQR 1.00)	< 0.05*
Cost of stay^c^ (dollars)	$109 486 (IQR 84 767)	$145 276 (IQR 104 065)	< 0.05*

*Note:* Outcomes reported as median (interquartile range) for non‐parametric continuous variables and percentage (number) for categorical variables.

^a^ Infectious complications include all‐cause infection, sepsis, or septic shock;

^b^ Length of stay reported in days;

^c^ Cost of stay reported in USD. *p* value considered significant < 0.05 (asterisk).

**Figure 4:**
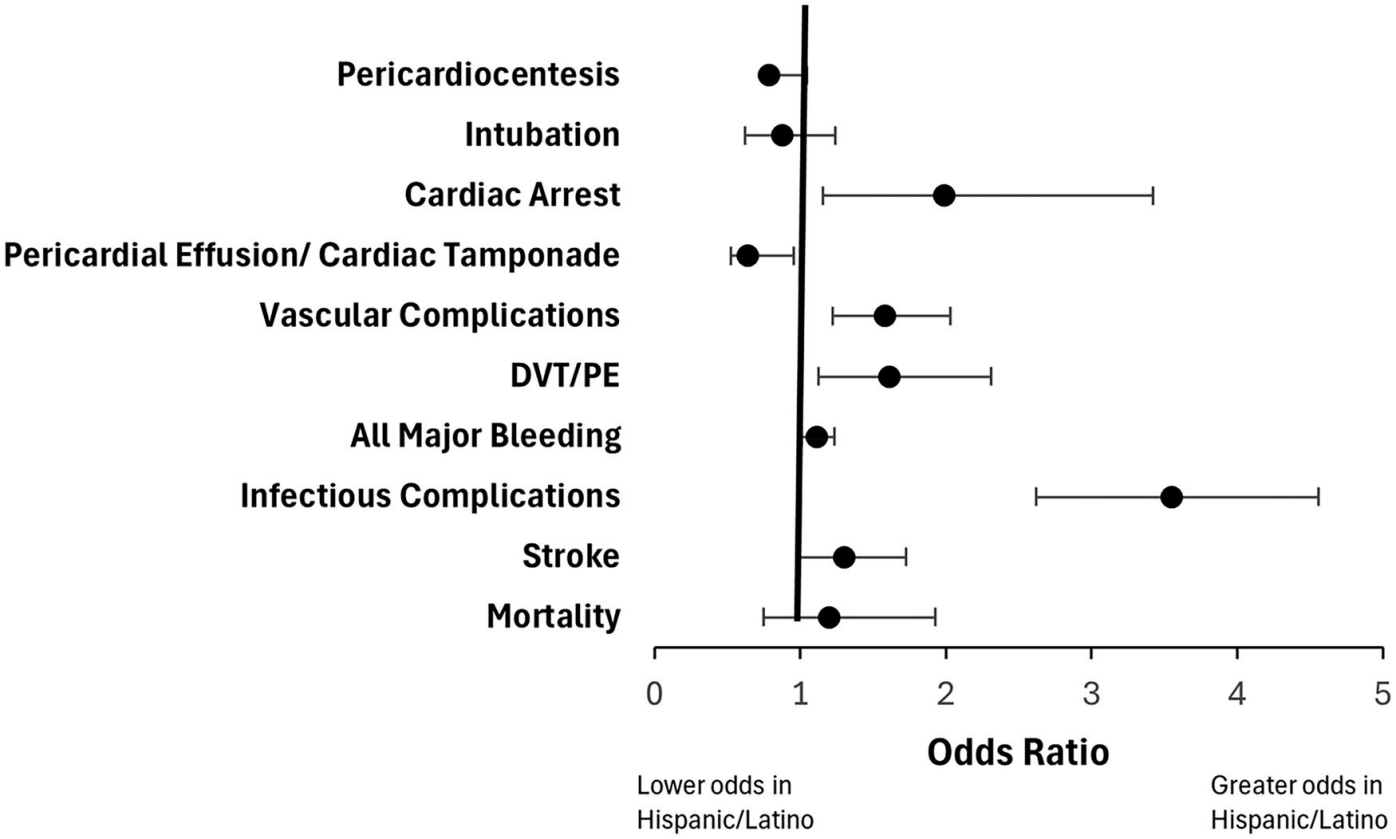
Adjusted odds ratios for outcomes for LAAO hospitalizations. Adjusted in‐hospital outcomes and events in Hispanic/Latin versus White patients hospitalized for LAAO reported as 95% confidence intervals. Values > 1 indicate higher odds in patients Hispanic/Latin patients.

Predetermined covariates and comorbidities were adjusted for by using a binary logistic regression model as presented in Figure 4. With the adjustments the Hispanic/Latino patients were found to have higher odds of infectious complications (aOR 3.54, 95% CI 2.62−4.55, *p* < 0.001) and vascular complications (aOR 1.57, 95% CI 1.22−2.03, *p* < 0.001). The higher odds of pericardial effusion/cardiac tamponade (aOR 0.64, 95% CI 0.52−0.95, *p* 0.03) and pericardiocentesis (aOR 0.785, 95% CI 0.59−1.04) in the white group, and cardiac arrest (aOR 1.99, 95% CI 1.15−3.42, *p* 0.46), vascular complications (aOR 1.579. 95% CI 1.22−2.03), DVT/PE (aOR 1.61, 95% CI 1.125−2.31), stroke (aOR 1.30, 95% CI 0.98–1.73, *p* 0.066), and mortality (aOR 1.20, 95% CI 0.75−1.92, *p* 0.09) in the Hispanic/Latino group were not statistically significant.

## 4 Discussion

We present a retrospective analysis of outcomes following LAAO in Hispanic/Latino patients in the United States. Previous studies have indicated that this population is at higher risk for bleeding and more frequent embolic strokes [11, 12]. Additionally, Hispanic/Latino patients admitted to the hospital for atrial fibrillation incur disproportionately higher costs for their stay compared to non‐Hispanic White and non‐Hispanic Black populations [13]. In our patient sample, chronic conditions such as diabetes mellitus, chronic kidney disease, stroke, and asthma had higher incidences among Hispanic/Latino individuals. Despite growing evidence supporting the efficacy of LAAO in preventing strokes and reducing mortality, Hispanic/Latino patients remain underrepresented in these studies and are less likely to undergo this procedure compared to their age‐matched non‐Hispanic White counterparts [14].

Here we demonstrate that despite being an overall younger group, and after adjusting for baseline characteristics, Hispanic/Latino patients undergoing LAAO still face higher adjusted odds of in‐hospital infectious and other vascular complications. White patients were found to have shorter hospital stays and lower associated cost. Additionally, our primary outcome measure of in‐hospital mortality was higher in Hispanic/Latino patients, although this difference was not statistically significant. We suspect the higher incidence of infectious and vascular complications are likely a multifactorial consequence of a higher comorbidity burden and differences in periprocedural care delivery.

The findings of higher complications and costs associated with LAAO in Hispanic/Latino patients have direct implications for clinical practice and contribute to the ongoing discussion about optimizing stroke prevention strategies in diverse patient populations. The study encourages health care providers to consider these disparities when making treatment decisions. It highlights the need for tailored interventions to help mitigate the increased risk and provide more equitable LAAO associated care.

## 5 Limitations

The relatively small number of Hispanic/Latino patients in the study reduces the generalizability of the findings to a broader population. This limitation makes it difficult to draw definitive conclusions about the Hispanic population's outcomes with this procedure. The study also did not include detailed echocardiographic or cardiac catheterization findings. These details are crucial for understanding the anatomical aspects of LAAO implantation, which could influence the decision to perform LAAO and its outcomes.

There is an unbalanced number of participants in each group (Hispanic vs. non‐Hispanic white), which may lead to biased sampling and affect the robustness of the comparative analysis. This imbalance can skew the results and limit the study's conclusions. Although the study mentions socioeconomic factors, it does not delve deeply into how these factors specifically impact access to care, treatment decisions, and outcomes [15, 16, 17]. More comprehensive socioeconomic data would provide a better understanding of the disparities observed. The study also found higher rates of mortality and complications in Hispanic patients but did not thoroughly explore the underlying reasons for these disparities. An in‐depth analysis on the factors contributing to these higher rates present a valuable opportunity for future research.

## 6 Conclusion

In Hispanic/Latino patients undergoing LAAO, both unadjusted and adjusted analyses reveal significantly higher odds of infectious and vascular complications compared to non‐Hispanic white patients. These findings highlight the importance of identifying and understanding racial and ethnic disparities in procedural outcomes. Characterizing and addressing these inequities is a critical step toward advancing equitable stroke prevention strategies and improving care delivery for patients with NVAF across diverse populations.

## Disclosure

The authors have nothing to report.

## Conflicts of Interest

The authors declare no conflicts of interest.

## Supporting information

LAAO Supportingl Material.

## Data Availability

All data used for this study are available in the National Inpatient Sample.

## References

[1] J. Garg , R. Kabra , R. Gopinathannair , et al., “State of the Art in Left Atrial Appendage Occlusion,” Journal of the American College of Cardiology EP 11, no. 3 (March 2025): 602–641, 10.1016/j.jacep.2024.10.024. 39797854

[2] U. A. Daimee , Y. Wang , F. A. Masoudi , et al., “Indications for Left Atrial Appendage Occlusion in the United States and Associated In‐Hospital Outcomes: Results From the NCDR Laao Registry,” Circulation. Cardiovascular Quality and Outcomes 15, no. 8 (2022): 008418, 10.1161/circoutcomes.121.008418. PMC9388561 35959677

[3] K. Bartus , F. T. Han , J. Bednarek , et al., “Percutaneous Left Atrial Appendage Suture Ligation Using the Lariat Device in Patients With Atrial Fibrillation,” Journal of the American College of Cardiology 62, no. 2 (2013): 108–118, 10.1016/j.jacc.2012.06.046. 23062528

[4] V. Y. Reddy , S. K. Doshi , S. Kar , et al., 5‐Year Outcomes After Left Atrial Appendage Closure From the PREVAIL and PROTECT AF Trials on behalf of the PREVAIL and PROTECT AF Investigators ABSTRACT BACKGROUND The PROTECT AF (WATCHMAN Left Atrial Appendage System for Embolic Protection in Patients with. 2017.

[5] J. Lopez , G. Duarte , R. A. Colombo , and N. E. Ibrahim , “Temporal Changes in Racial and Ethnic Disparities in the Utilization of Left Atrial Appendage Occlusion in the United States,” The American Journal of Cardiology 204 (2023): 53–63, 10.1016/j.amjcard.2023.07.040. 37536205

[6] A. Khedagi , F. Ugowe , and L. R. Jackson . Incidence and Prevalence of Atrial Fibrillation in Latinos: What's New Since the Study in Latinos (SOL),” In Current Cardiology Reports (Springer, 2023), 901–906, 10.1007/s11886-023-01910-w. 37421552

[7] D. S. Modaff and J. M. Wright , “Determinants and Disparities in Oral Anticoagulation Prescription: We Are Far From the PINNACLE of care,” In Heart Rhythm O2 (Elsevier B.V., 2023), 169–170, 10.1016/j.hroo.2022.12.007. PMC10041079 36993912

[8] U. A. Daimee , Y. Wang , F. A. Masoudi , et al., “Indications for Left Atrial Appendage Occlusion in the United States and Associated In‐Hospital Outcomes: Results From the NCDR LAAO Registry,” Circulation. Cardiovascular Quality and Outcomes 15, no. 8 (2022): 008418, 10.1161/CIRCOUTCOMES.121.008418. PMC9388561 35959677

[9] D. J. Daly , U. R. Essien , M. G. del Carmen , et al., “Race, Ethnicity, Sex, and Socioeconomic Disparities in Anticoagulation for Atrial Fibrillation: A Narrative Review of Contemporary Literature,” Journal of the National Medical Association 115 (2023): 290–297, 10.1016/j.jnma.2023.02.008. 36882341 PMC11333120

[10] J. V. Freeman , P. Varosy , M. J. Price , et al., “The NCDR Left Atrial Appendage Occlusion Registry,” Journal of the American College of Cardiology 75, no. 13 (2020): 1503–1518, 10.1016/j.jacc.2019.12.040. 32238316 PMC7205034

[11] H. Gardener , R. L. Sacco , T. Rundek , V. Battistella , Y. K. Cheung , and M. S. V. Elkind , “Race and Ethnic Disparities in Stroke Incidence in the Northern Manhattan Study,” Stroke 51, no. 4 (2020): 1064–1069, 10.1161/STROKEAHA.119.028806. 32078475 PMC7093213

[12] P. Calvert , K. Tamirisa , A. Al‐Ahmad , G. Y. H. Lip , and D. Gupta , “Racial and Ethnic Disparities in Stroke Prevention for Atrial Fibrillation,” American Journal of Medicine 136, no. 3 (2023): 225–233, 10.1016/j.amjmed.2022.11.009. 36495932

[13] L. Alhuneafat , A. Jabri , I. G. Poornima , et al., “Ethnic and Racial Disparities in Resource Utilization and In‐hospital Outcomes Among Those Admitted for Atrial Fibrillation: A National Analysis,” In Current Problems in Cardiology (Elsevier Inc, 2022), 10.1016/j.cpcardiol.2022.101365. 36031016

[14] J. A. Joglar , M. K. Chung , A. L. Armbruster , et al., “2023 ACC/AHA/ACCP/HRS Guideline for the Diagnosis and Management of Atrial Fibrillation: A Report of the American College of Cardiology/American Heart Association Joint Committee on Clinical Practice Guidelines,” In Circulation (Lippincott Williams and Wilkins, 2024), E1–E156, 10.1161/CIR.0000000000001193. PMC11095842 38033089

[15] S. A. Tang and S. K. Doshi , “Left Atrial Appendage Occlusion: Practice Makes Perfect?,” In JACC: Cardiovascular Interventions (Elsevier Inc, 2022), 962–964, 10.1016/j.jcin.2022.02.032. 35512919

[16] T. Nagasaka and M. Nakamura , “Left Atrial Appendage Closure: A Narrative Review,” In Cardiology and Therapy (Adis, 2023), 615–635, 10.1007/s40119-023-00337-2. PMC10704009 37938523

[17] J. Saw , D. R. Holmes , J. L. Cavalcante , et al., “Scai/Hrs Expert Consensus Statement on Transcatheter Left Atrial Appendage Closure,” Heart rhythm: The Official Journal of the Heart Rhythm Society 20, no. 5 (2023): e1–e16, 10.1016/j.hrthm.2023.01.007. 36990925

