# Supplementary material for: Outcomes of Left Atrial Appendage Occlusion in Hispanic/Latino Patients: Insights From the National Inpatient Sample
Source: Clin Cardiol. 2025 May 14;48(5):e70152. doi: 10.1002/clc.70152 (PMC12076124; doi:10.1002/clc.70152)
Supplement: Supplementary file 1 — LAAO Supportingl Material. [file CLC-48-e70152-s001.docx]

**Supplemental Material**

The National Inpatient Sample (NIS) compiles data on inpatient hospitalizations from 47 participating states and the District of Columbia. Serving as the largest all-payer inpatient database, it represents approximately a 20% sample of U.S. hospitalizations from community hospitals—which are general or specialized short-term hospitals, excluding rehabilitation and long-term acute care facilities. Each year, over 1,000 hospitals contribute data, accounting for about 7 million discharges annually. The NIS encompasses more than 97% of the U.S. population. To ensure privacy, patient, hospital, and state-level identifiers are omitted from the database. Institutional review board approval was not required because all information in the NIS is de-identified. Discharge weights are provided with each record to generate national estimates, and all data were weighted according to NIS recommendations. Prior to October 1, 2015, U.S. hospital administrative data were coded using the International Classification of Diseases, Ninth Revision, Clinical Modification/Procedure Coding System (ICD-9-CM/PCS). From the last quarter of 2015 onward, the NIS data are based on ICD-10-CM/PCS codes. These coding transitions are reflected in the structure of the NIS.

In this study, binary logistic regression models were adjusted for various factors including sex, elective admission status, diabetes mellitus, chronic kidney disease, hemodialysis, previous heart failure, tobacco use, substance abuse, anemia, asthma, chronic obstructive pulmonary disease (COPD), hyperlipidemia, and obstructive sleep apnea.

**Supplemental Table 1:** Codes used to identify LAAO hospitalizations were exclusively applied to the procedural section of the NIS database.

| **Variable** | **ICD 9 and 10 Codes** |
| --- | --- |
| Left atrial appendage occlusion | 02L73CK, 02L73DK, 02L73ZK, 02L74CK, 02L74DK, 02L74ZK, 3790 |

**Supplemental Table 2:** ICD-9-CM/PCS and ICD-10-CM/PCS codes for comorbidities and LAAO complications.

| **Variables** | **ICD-9 and ICD-10 codes** |
| --- | --- |
| Hypertension | 401.X, 402.X, 405.X, I10, I11.X, I12.X, I15.X |
| Diabetes Mellitus | 250.X, E08.X, E09.X, E10.X, E11.X, E12.X, E13.X, E14.X |
| Coronary Artery Disease | 412.X, 414.0X, 414.2, 414.3, 414.4, 414.8, 414.9, I25.1X, I25.2, I25.5, I25.6, I25.8X, I25.9 |
| History of Myocardial Infarctions | 412, I25.2 |
| Chronic Kidney Disease | 585.X, N18.X |
| Renal Replacement Therapy with Hemodialysis | 39.95. 5A1D00Z, 5A1D60Z, 5A1D70Z 5A1D80Z, 5A1D90Z |
| History of Heart Failure | 40201, 40211, 40291, 40401, 40403, 40411, 40413, 40491, 40493, I110, I130, I132, 39891, 4254, 4255, 4256, 4257, 4258, 4259, I099, I255, I420, I425, I426, I427, I428, I429, P290, I43 |
| Stroke History | 438.X, I69.X, V12.54, Z86.73 |
| Tobacco | 305.1, 989.84, V15.82, F17.X, T65.2X, Z72.0, Z87.891 |
| Alcohol | 291.1, 291.2, 291.3, 291.5, 291.6, 291.7, 291.81, 291.89, 291.9, 265.2, 303.00, 303.90, 303.91, 303.92, 303.93, 305.00, 305.01, 305.02, 305.03, 357.5, 425.5, 535.3, 571.0, 571.1, 571.2, 571.3, 980.X, E52, F10.X, G62.1, I42.6, K29.2, K70.0, K70.3, K70.9, T51.X, V11.3, Z50.2, Z71.4, Z72.1, |
| Substance abuse | 292.X, 304.X, 305.2, 305.3, 305.4, 305.5, 305.6, 305.7, 305.8, 305.9, F11.X, F12.X, F13.X, F14.X, F15.X, F16.X, F18.X, F19.X, V65.42, Z71.5, Z72.2 |
| Anemia | 280.X, 281.X, D50.X, D51.X, D52.X, D53.X |
| Asthma | 493.X, J45.X |
| Chronic Obstructive Pulmonary Disease | 490, 491.X, 492.X, 496, J40, J41.X, J42, J43.X, J44.X |
| Obesity | 278.00, 278.01, 278.03, E66.01, E66.09, E66.1, E66.2, E66.8, E66.9 |
| Peripheral Artery disease | 443.9, 440.X, I70.X, I73.9 |
| Obstructive Sleep Apnea | 78057, 32720, 32723, 32729, 78051, 78603, 78053, 32700, 32726, G4730, G4733, G4739, R0681 |
| Dyslipidemia | 272.X, E78.X |
| Pericardial Effusion/ Tamponade | I314, 4233, 4238 |
| Cardiac Arrest | 4275, I46.X |
| Stroke Complications | 430, I60.X, 431, 432.9, I61.X, 432.X, I62.X, 433.X, 434.X, 435.X, 436, 432.9, I63.X |
| Infectious Complications | 038.X, 995.91, 995.92, A40.X, A41.X, R65.2, 785.52, 998.02, T81.12, 998.51, 998.59, T814XXA, 996.60, 996.61, T826XXA |
| All Major Bleeding | 998.11, 998.12, I97.621, I97.630, I97.631, I97.638, I97.410, I97.411, I97.418, I97.42, I97.410, I97.411, I97.418, I97.42, I97.610, I97.611, I97.618, I97.620, 568.81, K66.1, 456.0, 456.20, 530.21, 530.7, 530.82, 531.00, 531.01, 531.20, 531.21, 531.40, 531.41, 531.60, 531.61, 532.00, 532.01, 532.20, 532.31, 532.40, 532.41, 532.60, 532.61, 533.00, 533.01, 533.20, 533.21, 533.40, 533.41, 533.60, 533.61, 534.00, 534.01, 534.20, 534.21, 534.40, 534.41, 534.60, 534.61, 535.01, 535.11, 535.21, 535.31, 535.41, 535.51, 535.61, 535.71, 537.83, 537.84, 562.02, 562.03, 562.12, 562.13, 569.3, 569.85, 569.86, 578.X, I85.01, I85.11, K22.11, K22.6, K22.8, K25.0, K25.2, K25.4, K25.6, K26.0, K26.2, K26.4, K26.6, K27.0, K27.2, K27.4, K27.6, K28.0, K28.2, K28.4, K28.6, K29.01, K29.21, K29.31, K29.41, K29.51, K29.61, K29.71, K29.81, K29.91, K31.811, K57.11, K57.13, K57.31, K57.33, K62.5, K55.21, K31.82, K63.81, K92.0, K92.1, K92.2, 596.7, 599.70, 599.71, N32.89, R31.9, R31.0, 786.30, 786.39, R04.2, R04.9, 784.7, R04.0, 459.0, R58 |
| DVT/PE | 415.11, 415.13, 415.19, 451.81, 451.9, 453.40, 453.41, 453.42, 453.8, 453.9, I82.491, I82.492, I82.493, I82.499, I82.409, I82.621, I82.622, I82.629, I82.401, I82.402, I82.623, I82.419, I824Y1, I82.4Y2, I82.4Y3, I82.403, I82.4Z1, I82.4Z2, I82.4Z3, I82.429, I82.A19, I82.890, I82.423, I82.439, I82.449, I82.413, I82.411, I82.412, I82.A11, I82.A12, I82.C11, I82.C12, I82.433, I82.443, I82.A13, I82.432, I82.441, I82.442, I82.C19, I82.421, I82.422, I82.C13, I82.431, I82.B19, I82.B13, I82.B11, I82.B12, I82.611, I82.612, I82.220, I82619, I82.613, I82.90, I82.4Y9, I82.4Z9, I82.649, I82.3, I81, I82.210, T8172XA, I82601, I82.602, I82.603, I97.89, I82.609, I82.451, I82.451, I82.452, I82.453, I82.459, I82.461, I82.462, I82.290, I26.99, I26.93, I26.02, I26.09 |
| Vascular Complications | 9982, 9992, 99771, 99772, 99779, 4470, I9751, I9752, T801XXA, T81710A, T81711A, T81718A, T8172XA, I770, 3956, 3931, 3941, 3949, 3952, 3957, 3959, 3979, 02QP0ZZ, 02QP3ZZ, 02QP4ZZ, 02QQ0ZZ, 02QQ3ZZ, 02QQ4ZZ, 02QR0ZZ, 02QR3ZZ, 02QR4ZZ, 02QW0ZZ, 02QW3ZZ, 02QW4ZZ, 02QX0ZZ, 02QX3ZZ, 02QX4ZZ, 02QS0ZZ, 02QS3ZZ, 02QS4ZZ, 02QV0ZZ, 02QV3ZZ, 02QV4ZZ, 900.X, 901.X, 902.X, 903.X, 904.X, 03Q.X, 04Q.X, 0W3.X, 02UP.X, 02UQ.X, 02U3.X, 02UR.X, 02US.X, 02UT,X, 02UV.X, 02UW.X, 03U.X, 03U.X, |
| Intubation | 0BH17EZ, 0BH13EZ, 0BH18EZ, 9604, 9605 |
